# The True Elements of Professional Character

**Published:** 1854-04

**Authors:** E. A. Arnold

**Affiliations:** Professor of Anatomy


### IOWA MEDICAL JOURNAL.

VOL. I. KEOKUK, IOWA, APRIL, 1854. NO. 9.

### ORIGINAL COMMUNICATIONS.

### *The True Elements of Professional Character. From an Address to the last Graduating Class of the Iowa Medical College.*

BY E. A. ARNOLD, M. D.
Professor of Anatomy.

* * * In view of the path which lies before you in the calling you have made your choice, I ask your attention to the consideration of the True Elements of Professional Character. Every man is prone to speak in praise of that calling, which by his own election, has become to him a life-work. Notwithstanding this, I am sure all will agree that the profession you have chosen, when clothed in its best and brightest belongings, has no superior among the occupations of men. Why then, do the workmen of our craft hold no higher rank among their fellow-laborers in the world? There can be no other reason than this—because so many go *unworthy* into the field. And whence proceeds such unworthiness? Simply and solely, for the reason, that so few seek to understand and attain the *true elements of professional character.*

It may be unjust that a cause really great and good should be so seriously injured by the standing of those who espouse it. But so it is, and none suffer so severely or on grounds so slight, as the science of medicine. Many have stood up as the ministers of the Most High

God, with feeble intellects and minds but too meagrely furnished—yea more, the purity of the sacerdotal robe has ofttimes been defiled by the wantonness, and debauchery of its wearers. Yet have the priesthood lost no whit of their power and respectability. Time and again have they, whose business is to expound the law, whose oath requires of them to "right the wrong and defend the right," made their profession a cloak for trickery, fraud and iniquity of every name. Nevertheless, no class of men share so liberally, in the honors and emoluments which the people can bestow. I speak these things, not in bitterness of spirit, but that I may the more fully impress upon your minds, the necessity laid upon you to strive throughout your future course, to elevate and purify *your* profession. If as a body, we would rise above our present position, how essential is it that we earnestly inquire after and strive to attain the true elements of professional character.

It is a common error among men to mistake *reputation* for *character*. This does not occur because there are lacking broad grounds of distinction between the two. Little reflection is needed to show those who so mistake, that they grasp after a shadow and neglect the substance. A reputation is easily gained. The fortunate recovery of a single patient may bring in a tide of practice. A wide spread medical reputation is not unfrequently obtained by the grossest empiricism and trickery. A metallic countenance, an oleaginous tongue and a low species of cunning are the only absolute requisites. I do not, by any means, despise that good fortune which sometimes follows a happy hit. This often comes to the well-deserving in time of need, like the gift of some kind fairy, raising the drooping spirits and reviving the sinking heart. I only caution you not to confound professional reputation with professional character, and tell you to beware how you run after the one and pass by the other, for in so doing you will make shipwreck of your brightest hopes. If now, in the beginning of your race, you make so fatal a mistake, you will find the way before you aught but pleasant; and as you approach the end with silvered hair and failing eye, you will find that you have striven in vain and lost your purest reward. Choose now, each one of you for himself, between the bubble I have shown you and the reality I am yet to present.

The life you are to lead, is, I am aware, no sinecure. But when the soldier has put on his armor, he must not shun the dangers of the battle. It will ill become you to shirk the trials and labors of the way. He who places before himself a high standard of excellence in any walk of life and hopes to attain thereto, must expect to *toil*, with patient, persevering, diligent faithfulness. If this be looked for in the ordinary occupations of life, how much more in those to whose care is entrusted the lives of their employers! But mark well the qualifying words I have used in connection with the term faithfulness. *Patient*, as the physician can never so thoroughly imbue himself with the precepts of the sage or the doctrines of a stoical philosophy, that he will remain always untouched by the petty vexations which constantly assail him. When annoyed by the unreasonable whims of the sick, when perplexed by the protean forms of disease, when tried by the obvious neglect of plain and wholesome directions, when teased by the impertinent queries of by-standers, he will find that homely old-fashioned virtue *patience*, worth more to him than all the maxims of Plato. *Persevering*, since that physician, who sometimes tires of his work or who makes the calls of duty give way to the calls of pleasure, should cease to hope for eminence or great excellence in his profession. Such an one is traveling a road filled with unbridged chasms and how can he expect, in the allotted time, safely to reach his journey's end!—His brightest hopes will be without foundation, his severest labors will be in vain unless tempered by a perseverance as unfailing as that sacred fire, which

"Though forever passed the days,
When God was worshiped in the blaze,
That from its mighty altars shone,
Though fled the priest, the votaries gone,
Still did the mighty flame burn on
Through chance and change, thro' good and ill,
Like its own God's eternal will,
Deep, constant, bright, unquenchable."

Moreover, if the physician is faithful, he must be a *laborer*. He must toil constantly, as well to furnish himself with the means of combatting disease, as in the use of those means when acquired. His practice must be supported by incessant study. In neither of these departments will his faithfulness be complete unless accompanied with the greatest *diligence*.

But there is an element, without which, the best of talents, the most unwearying patience, the most persevering diligence and the most faithful study will fail to make the physician successful. This is *sound practical judgment*. How many with the fairest prospects and fullest assurances of success, which good abilities and first rate acquirements can give, go forth into the world but to meet with bitter disappointment! This ensues because some important thing is lacking, and I hesitate not to say, that there is no more frequent cause of failure among those who have faithfully prepared themselves for their work, than the want of this quality. If the physician have not that judgment which shall teach him "when to act and when to forbear," rightly to choose and properly to apply his remedies, his endeavors must prove often futile or rather his success will be as frequently the result of chance as of scientific effort. He may be a perfect encyclopedia of medical knowledge. His mind may be a vast storehouse of scientific information. He may be the wonder and envy of his professional brethren, for the variety and extent of his attainments, and yet if sound practical judgment be wanting, he will be but a child at the bed-side of the sick. The key stone is wanting in an arch which would otherwise be a model of beauty and strength. The most important link is left out of a chain which, if complete, no power could break. Whoever else is excluded, in the season of distress, the physician must be allowed free entrance. In his passings to and fro, he must see and hear much that none others are permitted to know. What a mine of secrets may thus be laid open to him by expressions dropped in the ravings of delirium, by words forced out in the agony of pain, by facts related to give the medical attendant a complete understanding of a case, by confessions voluntarily made to one who has gained the confidence. Thus is the physician made the repository of his patient's private histories including much that, under other circumstances, would be most carefully hidden from the eyes of men. Hence the necessity of that *prudence* which should lead the disciples of our art to keep vigilant watch over every word they may utter. There is no principle more clearly laid down or more fully recognized in the code of medical ethics than this. I pronounce that man unworthy of the confidence of society or the countenance of the medical public, who going forth from the sick room, will allow himself to speak of scenes he may have witnessed or conversations he may have held by the bed-side of a patient. Growing out of this prudence and coupled with it, should be the power of *self-control.* Often tempted and sorely tried, how needful is it that the physician should have all his appetites, passions and feelings in complete subjection to his will and his will governed by high and righteous principle!

The relations which the physician sustains to community, should moreover, make him a *strictly honest* man. This is a self-evident truth and should need no demonstration. But the error is so common to mistake reputation for character, that I cannot refrain from pointing out the bearing which a lack of honesty has upon the character of a medical man. Hence, I say that however great his ability, he should never be allowed freely to enter the family circle or be admitted to those intimate relations in which a physician must stand, if he is expected faithfully to perform his professional duties in the hour of affliction. If he is not possessed of that purity of principle, which will make him perfectly honorable in the minor affairs of life, who will guarantee his honesty in those higher interests, where he is constantly surrounded by greater trials and more pressing temptations? These considerations call aloud upon every one entering this walk of life, to maintain, under all circumstances, a character not only free from reproach, but above all suspicion.

Thus far, I have spoken of those qualities, which may be considered necessary to success in any calling. Let me call your attention to some others which add peculiar beauty and glory to the character of the physician. There are some who assume the garb of your profession and render it rough and unseemly by rude and ungracious behaviour. While striving to mitigate the sufferings of the body, they aggravate the sorrows of the heart by harshness and incivility. In other words, they degrade their vocation by uncouth, uncourteous manners. How becoming then, and indispensable in the physician is a kind and gentlemanly deportment. I am not an admirer of the affectation or sickening airs of the exquisite—but he who does not endeavor so far to clothe himself with the attributes of the true gentleman, as never to give utterance to unkind or unfeeling expressions, at the bed side of the humblest patient, has no proper appreciation of professional character. He wears as a coarse and filthy habit what might be to him a robe of honor.

In a letter received from my father, during the early part of my studies, among other parental advice, I found these words: "Cultivate your heart." I repeat them on this occasion, because they contain a sentiment, which if I failed to inculcate upon you, I should come short of my duty. I have no fear but that in the course of your practice, you will have fully impressed upon you the necessity of striving to improve your minds. *Competition* will compel you to sharpen your intellects. *Ambition* will prompt you to increase your stock of knowledge. But while you thus pursue the cultivation of your minds you may dwarf the finer feelings of the soul or allow them to become calloused and wither away. After a certain position has been reached—when you have obtained enough of what may be called "paying practice," to keep you busy, selfishness or avarice may tempt you to neglect those calls from which you can expect no compensation. There is danger that in the tide of prosperity, you may be led to put off the suffering poor, that you may be induced to refuse your services to the sorrowing children of want. As you esteem the honor of your profession, as you value the highest reward which can follow the faithful performance of your duty, I warn you, never to turn away your feet from the abodes of misery, however humble or poverty-stricken they may be. Never lose sight of the brighter recompense that awaits those acts of kindness and mercy which should always mark the course of the true physician. To every one who desires to know the full dignity and glory of his profession, let the saying of that eminent man be a constant watchword through life. To those who were receiving instruction of him, he remarked: "The poor are my best patients, for God is their paymaster."

But on this occasion, I cannot refrain from expressing to you the valueless character of all things and all human attainments, except as they are viewed ultimately, in relation to a higher and holier state of existence. I am impelled to this by the consideration of an idea, which I know exists in the minds of many, that the study of medicine has a tendency to render men insensible to the claims of religion. Not unfrequently have I heard the opinion expressed that physicians are, as a class, skeptical, infidel and even atheistic in their views. Particularly is it said to be the case with those who devote themselves to the study of that branch, which it has been my duty to place before you. In my own experience and among my medical acquaintances, I have found this so far from the truth, that I do not hesitate to pronounce such an assertion utterly groundless. He who becomes so enthusiastic in his career as a medical man, as to make the shell annihilate the kernel, must either prefer the lower sympathies of the animal or violently doom himself to the desolateness of a deserted dwelling, where he might have the higher and refining fellowships of human and immortal spirits. Everything earthly derives its excellency and glory from its connection with a soul. Bear me witness then, that while we have been mainly occupied with the exterior anatomy of man, I have not failed to remind you of the inner man of the spirit, whose character and fearful relations, we leave you to settle with the clergy and your God.

*Gentlemen*—The days of your pupilage are now numbered with the past. Students you have been called while here—let not the same term be inapplicable to you in your future life. Go forth to your several stations, determined to perform your whole duty. Let this day be to you the commencement of a professional career, marked thoughout its course, by diligent, faithful study and careful, judicious practice. Be mindful also of those high and noble attributes which add such lustre to successful practice. As you depart hence, you carry with you our sincere, heartfelt wishes for your perfect success. We could not, if we would, withhold from you our warmest sympathies, since in your progress and future destiny, are bound up those seeds, which must ripen into honor and glory or shame and disgrace. As guardians of that Institution upon which must be reflected the influence of your well or ill-doing, we shall watch with anxiety your every step. Oh! let your course be such that we may behold you with satisfaction and be enabled to say with feelings of pride, that we were instrumental in guiding you on your way. Wherever you go, our eyes shall follow you and our hearts be with you to the end. May the richest blessings of Providence be ever in attendance on your well-directed efforts, and lead you to eminence and honor!

And now, as our sometime disciples, henceforward our professional brethren, we bid you an affectionate *farewell.*

